# Antitumor Activity of Pembrolizumab in Biomarker-Unselected Patients With Recurrent and/or Metastatic Head and Neck Squamous Cell Carcinoma: Results From the Phase Ib KEYNOTE-012 Expansion Cohort

**DOI:** 10.1200/JCO.2016.68.1478

**Published:** 2016-09-19

**Authors:** Laura Q.M. Chow, Robert Haddad, Shilpa Gupta, Amit Mahipal, Ranee Mehra, Makoto Tahara, Raanan Berger, Joseph Paul Eder, Barbara Burtness, Se-Hoon Lee, Bhumsuk Keam, Hyunseok Kang, Kei Muro, Jared Weiss, Ravit Geva, Chia-Chi Lin, Hyun Cheol Chung, Amy Meister, Marisa Dolled-Filhart, Kumudu Pathiraja, Jonathan D. Cheng, Tanguy Y. Seiwert

**Affiliations:** Laura Q.M. Chow, University of Washington, Seattle Cancer Care Alliance, Seattle, WA; Robert Haddad, Dana-Farber Cancer Institute, Boston, MA; Shilpa Gupta and Amit Mahipal, H. Lee Moffitt Cancer Center and Research Institute, Tampa, FL; Ranee Mehra, Fox Chase Cancer Center, Philadelphia, PA; Joseph Paul Eder and Barbara Burtness, Yale University Cancer Center, New Haven, CT; Hyunseok Kang, Johns Hopkins University, Baltimore, MD; Jared Weiss, Lineberger Comprehensive Cancer Center at the University of North Carolina, Chapel Hill, NC; Amy Meister, Marisa Dolled-Filhart, Kumudu Pathiraja, and Jonathan D. Cheng, Merck, Kenilworth, NJ; Tanguy Y. Seiwert, The University of Chicago, Chicago, IL; Makoto Tahara, National Cancer Center Hospital East, Kashiwa; Kei Muro, Aichi Cancer Center Hospital, Nagoya, Japan; Raanan Berger, Sheba Medical Center, Tel Hashomer; Ravit Geva, Sourasky Medical Center, Tel-Aviv, Israel; Se-Hoon Lee and Bhumsuk Keam, Seoul National University Hospital; Hyun Cheol Chung, Yonsei University College of Medicine, Seoul, Korea; and Chia-Chi Lin, National Taiwan University Hospital, Taipei, Taiwan.

## Abstract

**Purpose:**

Treatment with pembrolizumab, an anti–programmed death-1 antibody, at 10 mg/kg administered once every 2 weeks, displayed durable antitumor activity in programmed death-ligand 1 (PD-L1) –positive recurrent and/or metastatic (R/M) head and neck squamous cell carcinoma (HNSCC) in the KEYNOTE-012 trial. Results from the expansion cohort, in which patients with HNSCC, irrespective of biomarker status, received a fixed dose of pembrolizumab at a less frequent dosing schedule, are reported.

**Patients and Methods:**

Patients with R/M HNSCC, irrespective of PD-L1 or human papillomavirus status, received pembrolizumab 200 mg intravenously once every 3 weeks. Imaging was performed every 8 weeks. Primary end points were overall response rate (ORR) per central imaging vendor (Response Evaluation Criteria in Solid Tumors v1.1) and safety. Secondary end points included progression-free survival, overall survival, and association of response and PD-L1 expression. Patients who received one or more doses of pembrolizumab were included in analyses.

**Results:**

Of 132 patients enrolled, median age was 60 years (range, 25 to 84 years), 83% were male, and 57% received two or more lines of therapy for R/M disease. ORR was 18% (95% CI, 12 to 26) by central imaging vendor and 20% (95% CI, 13 to 28) by investigator review. Median duration of response was not reached (range, ≥ 2 to ≥ 11 months). Six-month progression-free survival and overall survival rates were 23% and 59%, respectively. By using tumor and immune cells, a statistically significant increase in ORR was observed for PD-L1–positive versus –negative patients (22% *v* 4%; *P* = .021). Treatment-related adverse events of any grade and grade ≥ 3 events occurred in 62% and 9% of patients, respectively.

**Conclusion:**

Fixed-dose pembrolizumab 200 mg administered once every 3 weeks was well tolerated and yielded a clinically meaningful ORR with evidence of durable responses, which supports further development of this regimen in patients with advanced HNSCC.

## INTRODUCTION

Head and neck squamous cell carcinoma (HNSCC) is the seventh most common cancer worldwide.^1^ In the United States, it is estimated there will be approximately 61,760 new cases of HNSCC and 13,190 deaths in 2016.^2^ Primary risk factors include tobacco smoking and alcohol consumption.^3^ Another risk factor for a growing subset of head and neck cancers is human papillomavirus (HPV) infection.^4^ Several differences among smoking- and alcohol-related versus HPV-associated HNSCC have been reported, including different patient characteristics, genomic profiles, and prognoses; for example, patients with HPV-associated HNSCC often have better outcomes than those with HPV-unrelated disease.^5^

A common first-line treatment regimen for recurrent and/or metastatic (R/M) HNSCC is the combination of cetuximab, platinum, and fluorouracil (EXTREME regimen), which has demonstrated a median overall survival (OS) of 10 months.^6^ Although this regimen has shown promising efficacy, patients who experience disease progression on first-line therapy or who are platinum refractory are seldom responsive to treatment, with response rates to second-line therapies—typically methotrexate, cetuximab, or taxanes—that range from 3% to 13%.^4,7^

HNSCC is generally associated with deficiencies of the immune system^8,9^; patients exhibit impaired natural killer cell activity, poor antigen-presenting function, low absolute lymphocyte counts,^10^ and mutations in genes that regulate inflammation.^9,11^ In the healthy immune system, programmed death-1 (PD-1) receptor functions as an immune checkpoint and is expressed primarily on the surface of activated CD4^+^ and CD8^+^ T cells.^12,13^ Engagement of PD-1 by either of its ligands, programmed death-ligand 1 or 2 (PD-L1 or PD-L2) results in inhibition of T-cell activation and limits the response to inflammation.^14,15^ In HNSCC and other solid tumors, tumor-infiltrating lymphocytes, and especially T helper 1 cells, activate interferon-mediated signaling and induce expression of PD-L1 on cells in the tumor environment, which protects tumor cells from tumor-directed immunity.^16,17^ Moreover, HNSCC tumor cells are known to exhibit high levels of PD-L1 expression.^17,18^ Preclinical studies indicate that blockade of the PD-1 and PD-L1 interaction enhances T-cell activation and inhibits tumor growth.^16,17,19^

Pembrolizumab, a highly selective humanized monoclonal immunoglobulin G4 antibody that blocks the interaction between PD-1 and its ligands, has demonstrated antitumor activity in multiple tumor types.^20-23^ Support for pembrolizumab in the treatment of R/M HNSCC has been described in the initial HNSCC cohort of the KEYNOTE-012 trial, which demonstrated clinical activity of pembrolizumab 10 mg/kg administered intravenously once every 2 weeks in patients with PD-L1–positive R/M HNSCC.^24^ Overall response rate (ORR) to this body weight–based dosing regimen was 18% (central imaging vendor review), with responses in 25% of patients who were HPV positive and 14% in those who were HPV negative. In that study, responses were durable (median, 53 weeks); median (95% CI) progression-free survival (PFS) and OS were 2 months (2 to 4 months) and 13 months (5 months to not-reached), respectively.

Recent studies that have used population pharmacokinetics and exposure-response models suggest that a lower, fixed dose of pembrolizumab (200 mg) and a less frequent administration schedule (once every 3 weeks) may be sufficient for target engagement and clinical activity.^25,26^ A fixed-dose regimen confers several advantages over body weight–based dosing, including safety, convenience, reduction of waste, and adherence. The aim of the current study was to report the safety and efficacy of a fixed-dose regimen in an all-comer population of patients with R/M HNSCC, regardless of PD-L1 or HPV status, from a larger HNSCC expansion cohort of the KEYNOTE-012 trial.

## PATIENTS AND METHODS

### Patients

The study was conducted in accordance with the Declaration of Helsinki and the International Conference on Harmonization Good Clinical Practice guidelines, and was approved by relevant regulatory and independent ethics committees. All patients provided written informed consent before study entry.

The KEYNOTE-012 trial was a phase Ib, multicenter, nonrandomized, multicohort study of pembrolizumab in patients with advanced solid tumors. Patients age ≥ 18 years with histologically or cytologically confirmed R/M HNSCC, measurable disease per RECIST (v1.1; Response Evaluation Criteria in Solid Tumors), Eastern Cooperative Oncology Group performance status of 0 or 1, and adequate organ function were eligible for enrollment in the expansion cohort. Patients had to provide an archival tumor sample at screening and newly obtained tumor biopsies before starting treatment and after 9 weeks of treatment; there were no eligibility criteria on the basis of biomarker expression—patients could be PD-L1 positive or negative and have HPV-associated or non–HPV-associated disease. HPV-associated disease refers to patients with a primary tumor location of the oropharynx who were considered by the site investigator to be HPV positive. Non–HPV-associated disease refers to patients with a non–HPV-associated oropharyngeal cancer and all patients with a primary tumor location outside of the oropharynx. There was no limit to the number of prior therapies; patients who were treatment naive were allowed. Patients who received previous treatments that specifically targeted T-cell costimulation or checkpoint pathways were excluded. Prior systemic immunosuppressive therapy had to be concluded within 7 days, chemotherapy or targeted small-molecule therapy within 2 weeks, and anticancer monoclonal antibody therapy within 4 weeks from the start of pembrolizumab. Patients with additional progressing malignancies, psychiatric or substance abuse disorders, HIV or active infection, CNS metastases, hepatitis B or C, or autoimmune disease were excluded.

### Study Design

Patients in this cohort received pembrolizumab 200 mg administered intravenously once every 3 weeks. Treatment continued for 24 months or until progressive disease (PD), intolerable toxicity, or investigator and/or patient decision to withdraw. Clinically stable patients with PD could remain on therapy until PD confirmation by a follow-up scan. Treatment response was assessed every 8 weeks by using computed tomography or magnetic resonance imaging. Patients who achieved complete response (CR) had the option of discontinuing therapy.

Adverse events (AEs) were monitored and graded according to National Cancer Institute Common Terminology Criteria for Adverse Events, version 4.0. Treatment was interrupted for grade 3 AEs or severe drug-related AEs until toxicity resolved to grade 0 to 1. Treatment was discontinued for grade 4 treatment-related AEs or if grade 3 AEs did not resolve within 12 weeks of the last treatment dose. Laboratory safety evaluations were performed within 10 days of the first study treatment and up to 72 hours before each dose.

The coprimary end points were safety and ORR per RECIST v1.1 by central imaging vendor review. The proportion of patients who achieved a CR, partial response (PR), and stable disease was determined. Secondary end points included ORR (RECIST v1.1 per investigator), PFS, OS, duration of response (DOR), and the correlation of response and PD-L1 expression.

The all-patients-as-treated population, which was defined as all patients who received one or more doses of pembrolizumab, was used for safety and efficacy analyses. An additional analysis of the primary efficacy end point was also conducted in all patients who received one or more doses of pembrolizumab, had measurable disease at baseline, and had one or more postbaseline scan or who discontinued treatment because of PD or treatment-related AE (full analysis set population).

### Biomarker Analysis

PD-L1 expression was retrospectively evaluated by using an immunohistochemistry (IHC) assay, an investigational version of the PD-L1 IHC 22C3 pharmDx assay (Dako, Carpinteria, CA) that uses the 22C3 (Merck, Kenilworth, NJ) anti–PD-L1 antibody. This assay was recently approved as the first PD-L1 IHC companion diagnostic for use in non–small-cell lung cancer (NSCLC) in the United States. The staining protocol used in this study was as described in the instructions for the approved commercial assay. The assay was used to determine PD-L1 expression on newly obtained or archival tumor samples. Two scoring methods were used to assess expression prevalence. One scoring method determined the percentage of tumor cells with membranous PD-L1 expression; details are provided in the scoring instructions for the approved commercial assay for NSCLC. A second scoring method took into account membranous PD-L1 staining on both tumor and mononuclear inflammatory cells. Both scores were measured on a scale from 0% to 100%; PD-L1 positivity was predefined as ≥ 1% using either of the two scoring methods.

### Statistical Analysis

With 100 evaluable patients, the study provided > 99% power to detect a difference of 15 percentage points in ORR under the null hypothesis of ORR = 5% with a type I error rate of 2.5% if the true ORR was 20%. Success for this hypothesis required at least 11 of 100 responses. Per protocol, the actual number of patients enrolled exceeded the target to ensure at least 100 evaluable patients.

Exact methods for binomial parameters were used to determine ORR per RECIST v1.1. Kaplan-Meier statistics were used to estimate PFS, OS, and DOR. Follow-up duration, which was defined as the time from enrollment to death or database cutoff, was calculated for all patients.

Logistic (ORR) or Cox (PFS and OS) proportional hazards regression one-sided testing was performed to assess the relationship between efficacy and PD-L1 expression measured by using tumor cells alone and a combination of tumor and immune cells. These analyses used centrally reviewed ORR, PFS, and OS.

## RESULTS

### Patients

**Fig 1.**
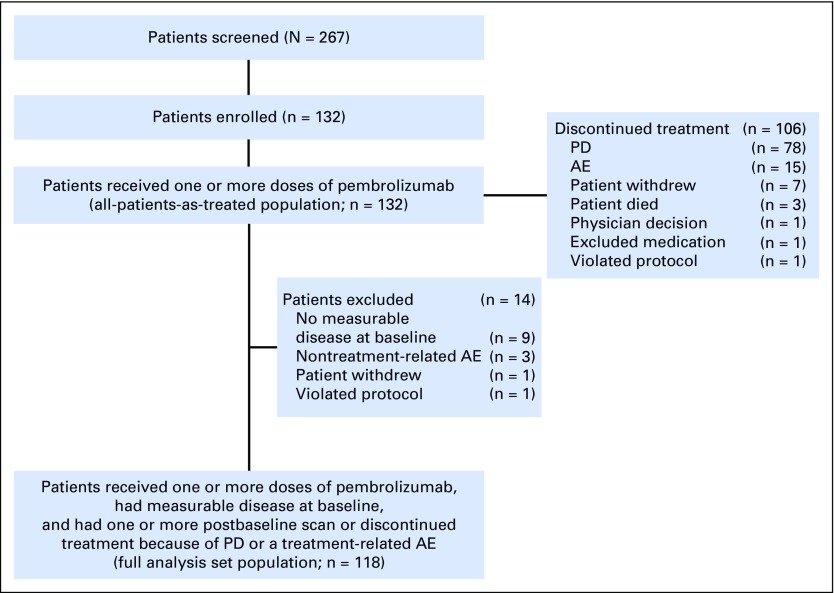
Consort diagram. Patient disposition. AE, adverse event; PD, progressive disease.

In total, 132 patients were enrolled between June 12, 2014, and October 8, 2014; the cutoff for data accrual was September 1, 2015. All enrolled patients received one or more doses of pembrolizumab—an all-patients-as-treated population—and were included in safety and efficacy analyses. The full analysis set population used for the primary efficacy end point included 118 patients (Fig 1). Twenty-eight patients (21%) had HPV-associated HNSCC and 104 (79%) had non–HPV-associated disease. Patients were a median of age 60 years (range, 25 to 84 years), and 110 (83%) were male (Table 1). Patients were heavily pretreated: 57% received two or more prior therapies for R/M disease. During the study, therapy was discontinued in 106 patients (80%) because of PD (n = 78), an AE (n = 15), physician or patient decision (n = 8), death (n = 3), need for medication exclusion (n = 1), and protocol violation (n = 1; Fig 1).

**Table 1.**
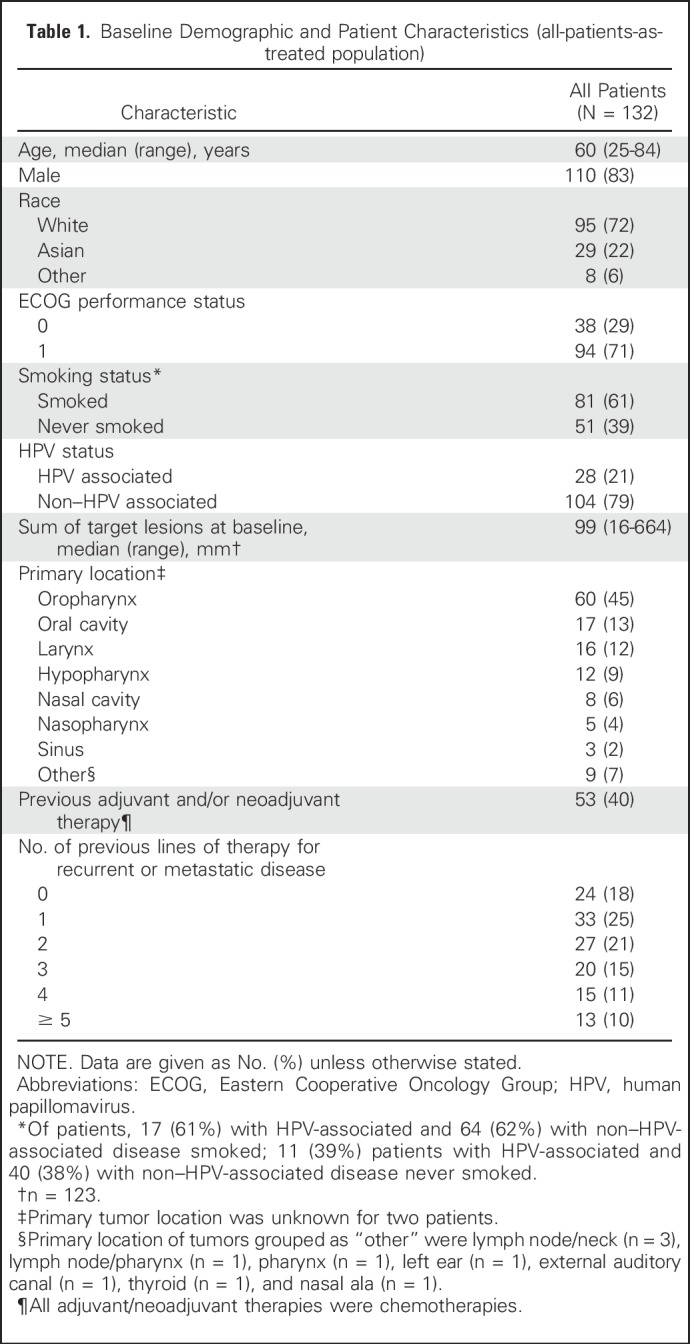
Baseline Demographic and Patient Characteristics (all-patients-as-treated population)

### Safety

Median number of days on pembrolizumab was 88 (range, 1 to 411 days). Treatment-related AEs occurred in 82 patients (62%; Table 2). The most frequently occurring treatment-related AEs of any grade included fatigue (n = 28), hypothyroidism (n = 14), and decreased appetite (n = 11). Twelve patients (9%) experienced a grade 3 or 4 treatment-related AE (Table 2), most commonly decreased appetite, facial swelling, and pneumonitis, which occurred in 2 patients each. There were no treatment-related deaths. Eight patients (6%) discontinued treatment because of treatment-related AEs, and 29 patients (22%) had one or more dose interruptions as a result of an AE. AEs of special interest because of immune-related etiology, regardless of causality, occurred in 26 patients (20%); most events were of grade 1 or 2 severity. Grade 3 immune-related AEs included pneumonitis (n = 2), diabetes mellitus (n = 1), decubitus ulcer (n = 1), colitis (n = 1), and drug-induced liver injury (n = 1); one grade 4 immune-related AE (diabetic ketoacidosis) occurred. Of these, two patients who experienced grade 3 pneumonitis, one patient with grade 3 colitis, and one patient with grade 2 interstitial lung disease had to discontinue study treatment.

**Table 2.**
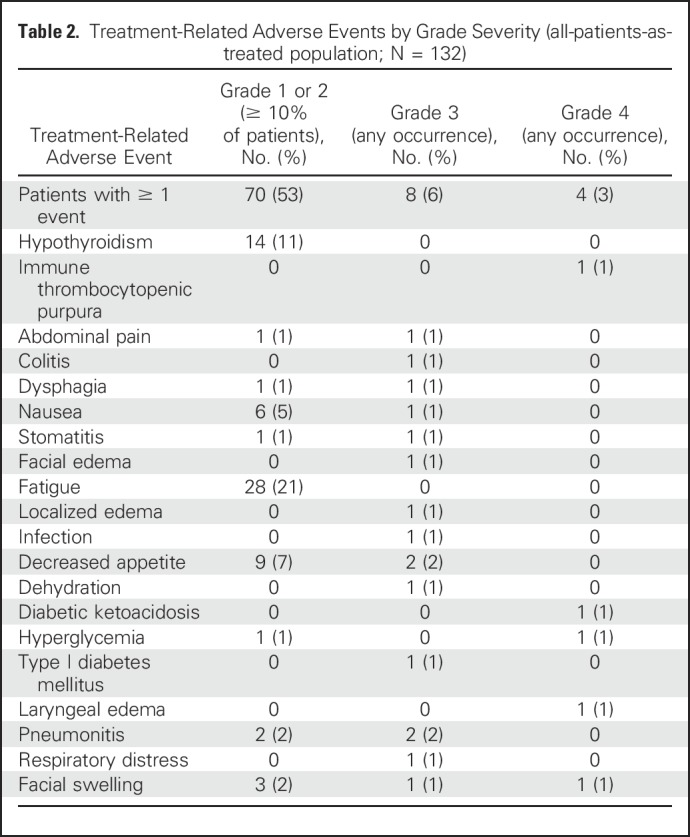
Treatment-Related Adverse Events by Grade Severity (all-patients-as-treated population; N = 132)

### Efficacy

**Fig 2.**
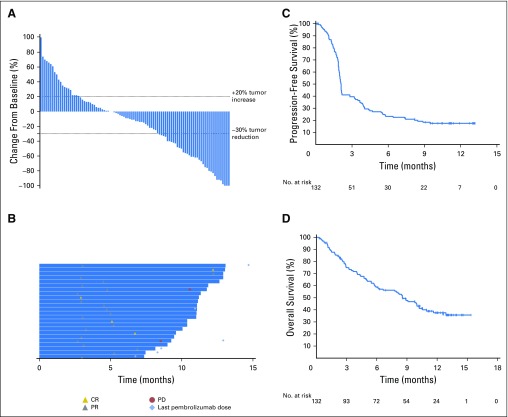
Efficacy of pembrolizumab on the basis of RECIST (v1.1; Response Evaluation Criteria in Solid Tumors) by central imaging vendor review. (A) Maximum percentage change from baseline in target lesions. Includes patients who had a comparable number of lesions between baseline and postbaseline scans (n = 96). (B) Treatment exposure and response duration in responders (all responders, n = 24). (C and D) Kaplan-Meier estimates of (C) progression-free survival and (D) overall survival (all-patients-as-treated population, N = 132). CR, complete response; PD, progressive disease; PR, partial response.

After a median follow-up duration of 9 months (interquartile range, 3 to 11 months), ORR in the all-patients-as-treated population was 18% (95% CI, 12% to 26%) by central imaging vendor review and 20% (95% CI, 13% to 28%) by investigator review (Table 3). An additional analysis of the primary end point using the full analysis set population yielded ORRs of 20% (95% CI, 13% to 29%) and 21% (95% CI, 14% to 29%) by central imaging vendor review and investigator review, respectively. In the all-patients-as-treated population by central review, 4 (3%) of 132 patients achieved a CR, 20 (15%) achieved a PR, 26 (20%) had stable disease, and 61 (46%) experienced disease progression. The total disease control rate, which was defined as the sum of CR, PR, and stable disease for ≥ 6 months, was 20%. No cases of pseudoprogression were observed in this cohort. The ORR was 32% (9 of 28 patients) and 14% (15 of 104 patients) among those with HPV-associated and non–HPV-associated disease, respectively (Table 3). When patients with non–HPV-associated disease were further evaluated by their tumor location in the oropharynx, oral cavity, hypopharynx, and larynx, ORR was 15% (11 of 76 patients). Patients with non–HPV-associated disease in other locations had an ORR of 14% (4 of 28 patients). A reduction in target lesion size of any amount was found in 61% of all patients (Fig 2A).

**Table 3.**
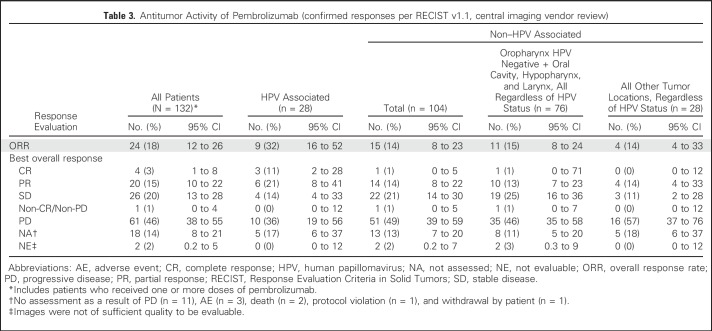
Antitumor Activity of Pembrolizumab (confirmed responses per RECIST v1.1, central imaging vendor review)

Median time to response was 2 months (range, 2 to 11 months). Observed responses were durable and persisted over several assessments, with a median DOR that was not reached (range, ≥ 2 to ≥ 11 months) at the data cutoff. At the time of writing, 26 patients remained on therapy, and 20 (83%) of 24 radiologic responses were ongoing (Fig 2B).

Median PFS was 2 months (95% CI, 2.0 to 2.2 months) and the PFS rate at 6 months was 23% (Fig 2C). The 6-month PFS rate in patients with HPV-associated and non–HPV-associated HNSCC was 37% and 20%, respectively. Median OS was 8 months (95% CI, 6 to 10 months). The 6-month OS rate was 59% in all patients (Fig 2D) and 70% and 56% in patients with HPV-associated and non–HPV-associated disease, respectively.

### Biomarker Analysis

**Fig 3.**
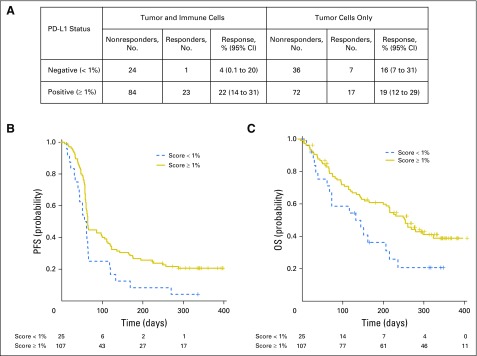
(A) Association of efficacy and programmed death-ligand 1 (PD-L1) expression. Overall response by PD-L1 expression in tumor and immune cells and tumor cells alone. (B and C) Kaplan-Meier estimates of (B) progression-free survival (PFS) and (C) overall survival (OS) on the basis of a positive expression cutoff of ≥ 1% in tumor and immune cells (all-patients-as-treated population, N = 132).

When PD-L1 expression analyses were restricted to only tumor cells, there was no statistically significant increase in the probability of response for patients with positive (≥ 1%) versus negative (< 1%) tumors (*P* = .348, one-sided test). Conversely, when immune cells were included in the scoring system, a statistically significant increase in the probability of response for positive (≥ 1%) versus negative (< 1%) patients was observed (*P* = .021, one-sided test). ORR in patients who were PD-L1 positive by tumor and immune cell scoring was 22%, whereas those who were negative had an ORR of 4% (Fig 3). Similarly, statistically significant differences for PFS and OS were observed when scoring took into account staining in both tumor and immune cells (PFS: *P* = .008; OS: *P* = .008, one-sided tests) but not tumor cells alone (PFS: *P* = .195; OS: *P* = .132, one-sided tests). Median OS times for patients who were PD-L1 positive versus negative by tumor and immune cell scoring were 303 days and 151 days, respectively (Fig 3).

## DISCUSSION

This study establishes the efficacy and safety of a fixed dose of pembrolizumab (200 mg) administered once every 3 weeks in patients with HNSCC. With an ORR of 18% by central imaging vendor review, an ORR of 20% by investigator review, an OS of 8 months, and a DOR that was not reached, the clinical benefit of pembrolizumab in biomarker-unselected patients with R/M HNSCC was similar to that seen in the initial cohort of patients who were PD-L1 positive (ORR, 18% by central imaging vendor review; median OS, 13 months; median DOR, 53 weeks).^24^ The response in this heavily pretreated population compares favorably with that observed with single-agent cetuximab (ORR, 13%; DOR, 4 months).^27^ In addition, median OS was on par with the longest median survival reported in patients with treatment-naive R/M HNSCC (10 months), which was achieved with a combination of platinum, cetuximab, and fluorouracil (EXTREME regimen),^6^ and similar to that observed with afatinib in patients with previously treated R/M disease (7 months).^28^ In contrast to the significant toxicity observed with the EXTREME regimen, pembrolizumab was well tolerated in this study. The safety profile in the expansion cohort was consistent with the safety profiles of pembrolizumab that were reported in the initial HNSCC cohort and in studies in melanoma and NSCLC.^21,22,25^

Approximately one half of all patients in this study with HNSCC of the oropharynx had HPV-associated disease, which has been associated with longer survival than non–HPV-associated HNSCC.^5^ Similar to the initial HNSCC cohort of the KEYNOTE-012 trial, a higher response to pembrolizumab was observed in patients with HPV-associated versus non–HPV-associated HNSCC.^24^ Of note, an ORR of 14% was consistently observed in patients with HPV-negative disease in both KEYNOTE-012 HNSCC cohorts, which suggests that those with non–HPV-associated R/M HNSCC—a group with particularly poor prognosis—may also benefit from pembrolizumab. This is not surprising given the emerging evidence that supports the use of PD-1–targeted therapies to treat both HPV-associated and non–HPV-associated HNSCC.^18,29^ Although HPV-associated and non–HPV-associated HNSCC differ in several ways, and the mechanisms that lead to PD-L1 expression may differ between the two, PD-L1 has been shown to be overexpressed in both settings. Whether there is a difference between PD-L1 expression levels in HNSCC on the basis of HPV association remains unclear given the current literature.

A limitation of this study is the lack of a consistent method used to determine HPV status. HPV association was determined by the site investigator by using the method of their choice; p16 IHC was used by the majority of sites. Whereas p16 IHC is a useful surrogate for HPV infection in oropharyngeal HNSCC, it has limited utility outside of the oropharynx where HPV is less prevalent.^30,31^ For that reason, patients with nonoropharyngeal HNSCC were considered to be HPV-negative regardless of p16 status. In addition, the number of patients with HPV-associated disease in this study was substantially less than the number with non–HPV-associated HNSCC. Response by HPV association should be interpreted with these limitations in mind.

On the basis of an evolving body of evidence in HNSCC and other solid tumors that suggest PD-L1 expression on tumor-infiltrating immune cells may contribute to clinical outcome,^32,33^ two scoring systems were used to determine PD-L1 expression: one that analyzed expression on only tumor cells and another that included both tumor and immune cells. Although we did not independently determine the contribution of each cell type, we have demonstrated that PD-L1 expression on immune cells significantly contributes to the predictability of response in HNSCC, as PD-L1 expression on tumor cells alone was not significantly correlated with response.

Results from the current study indicate that the less frequent, fixed dose of pembrolizumab is tolerable and provides comparable antitumor activity with pembrolizumab administered in a body weight–based dosing regimen. Patients responded to pembrolizumab regardless of HPV status, and PD-L1 expression on tumor and immune cells was found to be associated with response. These findings support ongoing phase II (KEYNOTE-055) and phase III (KEYNOTE-040 and KEYNOTE-048) studies of the fixed-dose regimen in patients with advanced HNSCC.
